# Injecting vascular endothelial growth factor into the temporomandibular joint induces osteoarthritis in mice

**DOI:** 10.1038/srep16244

**Published:** 2015-11-04

**Authors:** Pei Shen, ZiXian Jiao, Ji Si Zheng, Wei Feng Xu, Shang Yong Zhang, An Qin, Chi Yang

**Affiliations:** 1 Department of Oral Surgery, Ninth People’s Hospital, College of Stomatology, Shanghai Jiao Tong University School of Medicine, Shanghai Key Laboratory of Stomatology & Shanghai Research Institute of Stomatology, Shanghai, People’s Republic of China; 2 Shanghai Key Laboratory of Orthopedic Implants, Department of Orthopedics, Shanghai Ninth People’s Hospital, Shanghai Jiao Tong University School of Medicine, Shanghai, People’s Republic of China

## Abstract

It is unclear whether vascular endothelial growth factor (VEGF) can initiate osteoarthritis (OA) in the temporomandibular joint (TMJ). In this study we evaluated the effects of intra-articular injection of exogenous VEGF in the TMJ in mice on the early stage. Forty-eight male Sprague-Dawley mice were equally divided into 3 groups. In the vegf group, the mice received an injection of VEGF solution (50 μL) in the TMJ once a week over a period of 4 weeks. In the sham group, the mice received an injection of saline (50 μL). The control group did not receive any injection. Four mice from each group were sacrificed at 1, 2, 4, and 8 weeks. Gradual prominent cartilage degeneration was observed in the vegf group. Additionally, this group showed higher expressions of metalloproteinase (MMP)-9, MMP-13, receptor activator of nuclear factor-kappa-B ligand (RANKL), and a higher number of apoptotic chondrocytes and VEGF receptor 2 (VEGFR2)-positive chondrocytes. Micro-computed tomography (CT) revealed prominent subchondral bone resorption in the vegf group, with a high number of osteoclasts in the subchondral bone. *In vitro* study demonstrated that VEGF can promote osteoclast differentiation. In conclusion, our study found that VEGF can initiate TMJ OA by destroying cartilage and subchondral bone.

Osteoarthritis (OA) is a common degenerative joint disease characterized by synovitis, cartilage degeneration, subchondral bone sclerosis, and osteophyte formation. The temporomandibular joint (TMJ), which is the only diarthrodial joint in the human jaw, can be affected by OA. In autopsies of young individuals and the elderly, degenerative changes in the TMJ were found in 28% and 50% of these individuals, respectively^1^. The clinical symptoms of TMJ OA include pain, a clicking sound from the jaw, limitations in keeping the mouth open, and facial asymmetry, which may seriously affect a patient’s quality of life^2^. The pathogenesis of TMJ OA has not yet been clarified.

In recent years, an increasing number of studies have focused on the molecular mechanisms of OA. Previous reports showed that angiogenesis (the formation of new blood vessels from preexisting vessels) might be involved in the progression of OA. Additionally, neo-angiogenesis was shown to damage cartilage and lead to chondrocyte death^3^,^4^. Vascular endothelial growth factor (VEGF) is a potent, pro-angiogenic growth factor that is expressed in synoviocytes and chondrocytes during cartilage growth. Both VEGF and its receptors have been detected within the superficial chondrocyte layer in growing articular cartilage^5^. However, VEGF expression is rarely detected in mature articular cartilage. Interestingly, VEGF and its receptors were detected in the chondrocytes of human osteoarthritic joints^3^,^6^,^7^. VEGF was shown to affect chondrocytic proliferation, apoptosis, and metabolism, leading to the release of metalloproteinases (MMPs), as well as other catabolic mediators that degrade the cartilage matrix^8^,^9^,^10^.

In the TMJ, VEGF is involved in articular cartilage destruction and TMJ OA development. Sato *et al.*^11^ reported that VEGF expression in the synovial fluid was 5-fold higher in TMJs with internal derangement than in healthy joints. Tanaka *et al.*^12^ and Shirakura *et al.*^13^ found high VEGF expression in the condylar cartilage of TMJ OA model rats, suggesting an important role of VEGF in the development of TMJ OA.

It is unclear whether VEGF can initiate TMJ OA-like changes, and the potential molecular mechanisms are unknown. Thus, in the present study, we evaluated the effects of intra-articular injection of exogenous VEGF in the TMJ in mice. We focused on the early changes in the condyle cartilage and subchondral bone of the TMJ to confirm whether VEGF can initiate TMJ OA.

## Results

### Cartilage changes

#### Histological cartilage changes

**Figure 1:**
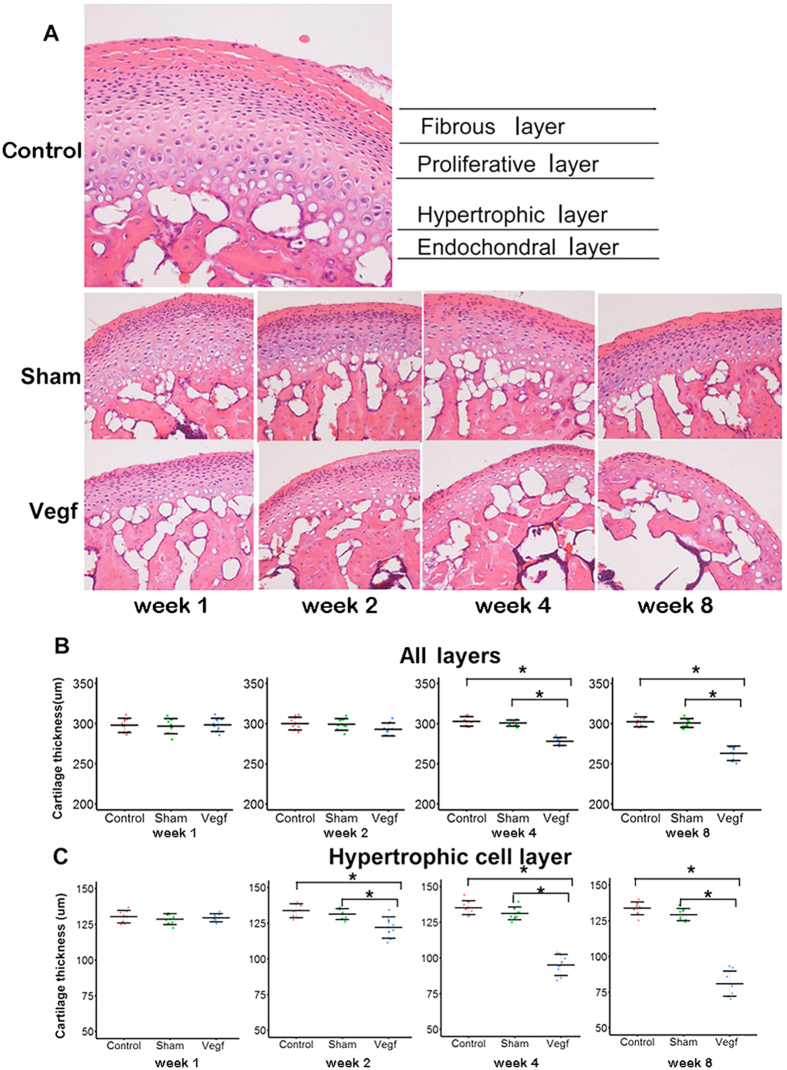
Thickness of the condyle cartilage in the control, sham, and vegf groups at weeks 1, 2, 4, and 8. (**A**) Central sagittal sections of the condyle are stained with hematoxylin and eosin (×200). All condylar cartilage layers, especially the hypertrophic layer, appear thinner after week 2 in the vegf group than in the sham and control groups. (**B,C**) Comparison of the total cartilage thickness and hypertrophic layer thickness between the groups. All condylar cartilage layers, especially the hypertrophic layer, are significantly thinner after week 2 in the vegf group than in the sham and control groups (*P < 0.05).

Hematoxylin and eosin (HE) staining showed that the surface of the condylar cartilage in the control group was intact and smooth. The condylar cartilage is made up fibrous, proliferative, hypertrophic, and endochondral ossification layers (Fig. 1A). Histological analysis showed that the morphology of the condylar cartilage was similar between the sham group and the control group at every time point. However, all condylar cartilage layers, especially the hypertrophic layer, were significantly thinner after week 2 in the vegf group than in the sham and control groups (Fig. 1B,C; P < 0.05), and the thickness of the layers further decreased as time progressed in the vegf group. Vacuolation and degeneration were also observed in the vegf group at weeks 4 and 8.

**Figure 2:**
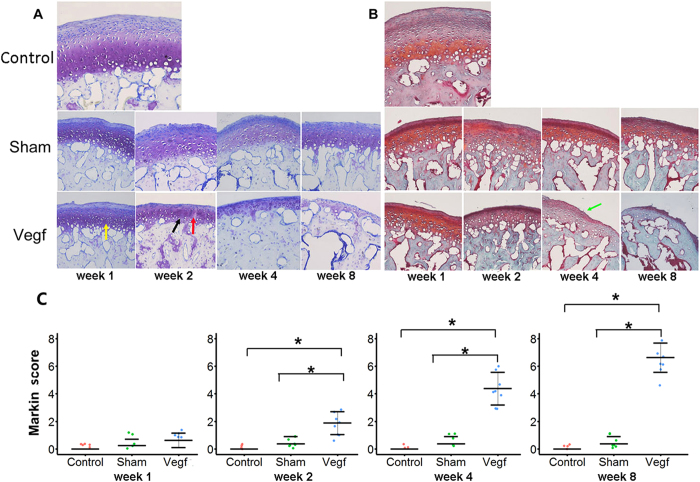
Degenerative changes in the condyle cartilage in the control, sham, and vegf groups at weeks 1, 2, 4, and 8. (**A**) Proteoglycan changes in the condyle observed with toluidine blue. (**B**) Safranin-O and fast green staining (×200). Gradual but pronounced proteoglycan loss is observed in the vegf group over time. Additionally, irregular arrangement of chondrocytes (yellow arrow), chondrocyte clusters (black arrow), an increase in the cell free area (red arrow), and fibrillation (green arrow) are only observed in the vegf group. (**C**) Comparison of the Mankin scores between the groups. The Mankin scores are significantly higher in the vegf group than in the sham and control groups from week 2 onwards (*P < 0.05).

On toluidine blue, and safranin-O and fast green staining, the condylar cartilage in the control group exhibited a rich and even distribution of proteoglycans, especially in the deep layers of the cartilage (Fig. 2A,B). A slight increase in proteoglycan was seen throughout the condylar cartilage after week 1 in the sham and vegf groups. However, proteoglycan normalized in the sham group by week 2, while a gradual but pronounced loss of proteoglycan was observed in the vegf group over time. Furthermore, the arrangement of chondrocytes was irregular after week 1 in the vegf group (Fig. 2A). Chondrocyte clusters, as well as an increase in the cell free area were observed after week 2 in the vegf group (Fig. 2A). At week 4, fibrillation was seen in the vegf group (Fig. 2B), and the loss of articular cartilage was evident at week 8. These changes were rarely observed in the sham group.

#### Mankin scoring of the cartilage

Morphological changes in the articular cartilage of the TMJ were scored according the modified Mankin scoring system. There were no significant differences in the scores between the sham and control groups at all time points. However, the scores were significantly higher (reflecting progressive degeneration of the articular cartilage) in the vegf group than in the sham and control groups from week 2 onwards (Fig. 2C; P < 0.05).

#### Metabolic changes in the cartilage and chondrocyte apoptosis

**Figure 3:**
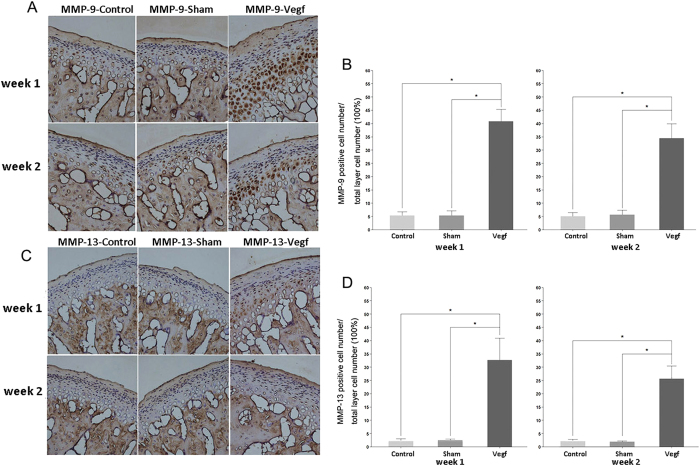
Expressions of MMP-9 and MMP-13 in the control, sham, and vegf groups at weeks 1 and 2. (**A,C**) Immunohistochemical staining for MMP-9 (**A**) and MMP-13 (**C**) at weeks 1 and 2. High numbers of MMP-9- and MMP-13-positive cells are observed in the vegf group. (**B,D**) Comparison of the percentages of MMP-9- (B) and MMP-13-positive cells (**D**) between the groups. The percentages of MMP-9- and MMP-13-positive cells are significantly higher in the vegf group than in the control and sham groups (*P < 0.05).

**Figure 4:**
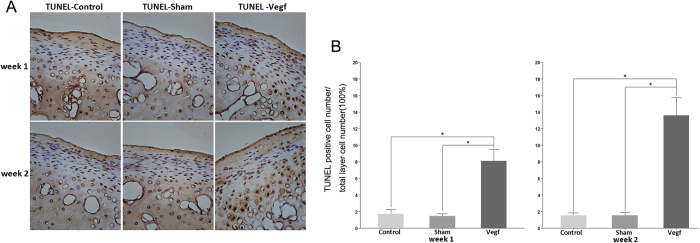
Chondrocyte apoptosis in the control, sham, and vegf groups at weeks 1 and 2. (**A**) Terminal dUTP nick-end labeling (TUNEL) staining at weeks 1 and 2. A high number of TUNEL-positive cells is observed in the vegf group. (**B**) Percentage of TUNEL-positive cells. The percentage of TUNEL-positive cells is significantly higher in the vegf group than in the control and sham groups (*P < 0.05).

In order to identify the early metabolic changes in the condylar cartilage of the TMJ after injecting VEGF in the articular cavity, immunohistochemical staining for MMP-9 and MMP-13, and terminal dUTP nick-end labeling (TUNEL) was performed in the specimens at weeks 1 and 2. MMP-9- and MMP-13-positive cells were distributed in the hypertrophic layer, while TUNEL-positive cells were distributed in all layers of the cartilage. Few MMP-9-, MMP-13-, and TUNEL-positive cells were identified in the cartilage in the control and sham groups, while many MMP-9-, MMP-13-, and TUNEL-positive cells were identified in the vegf group (Figs 3A,C and 4A). The percentages of MMP-9-, MMP-13-, and TUNEL-positive cells were significantly higher in the vegf group than in the control and sham groups (Figs 3C,D and 4B; P < 0.05).

### Subchondral bone changes

#### Micro-computed tomography (CT) analysis

**Figure 5:**
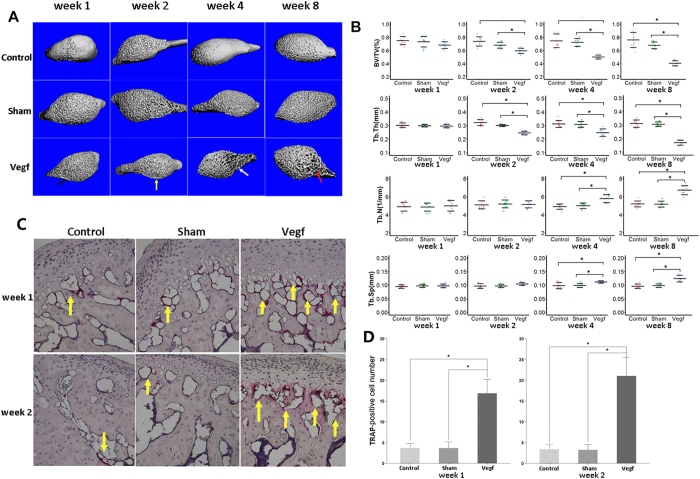
Subchondral bone destruction and resorption in the control, sham, and vegf groups at weeks 1, 2, 4, and 8. (**A**) Three-dimensional images of the temporomandibular joint condyle. Subchondral bone lesions are seen in the vegf group at weeks 1 and 2 (black and yellow arrows, respectively), while bone lesions accompanied with local sclerosis are seen in the vegf group at weeks 4 and 8 (white and red arrows, respectively). (**B**) Comparison of subchondral bone features using micro-CT scanning. The bone volume fraction (BV/TV) and trabecular thickness (Tb.Th) are significantly lower in the vegf group than in the control and sham groups from week 2 onwards, and the trabecular number (Tb.N) and trabecular separation (Tb.Sp) are significantly higher in the vegf group than in the control and sham groups from week 4 onwards (*P < 0.05). (**C**) A high number of tartrate-resistant acid phosphatase (TRAP)-positive osteoclasts is seen in the vegf group (yellow arrows). (**D**) Comparison of the number of TRAP-positive osteoclasts. The number of TRAP-positive osteoclasts is significantly higher in the vegf group than in the control and sham groups (*P < 0.05).

Micro-CT analysis revealed that the subchondral bone of the condyle was evenly aligned in the sham and control groups at all time points. However, progressive osteoarthritic changes were observed in all joint tissues in the vegf group. In the vegf group, small, subchondral bone lesions were noted in approximately 38% of the joints analyzed at week 1 (Fig. 5A), and the lesions enlarged over time. Additionally, at week 2, 75% of the joints had local bone lesions (Fig. 5A). Moreover, bone lesions, accompanied with local sclerosis, were observed at weeks 4 and 8 (Fig. 5A).

In the micro-CT analysis, there were no significant differences in the microstructural parameters of the subchondral bone between the sham and control groups at all time points. However, the bone volume fraction (BV/TV) and trabecular thickness (Tb.Th) were significantly lower in the vegf group than in the control and sham groups from week 2 onwards, and the trabecular number (Tb.N) and trabecular separation (Tb.Sp) were significantly higher in the vegf group than in the control and sham groups from week 4 onwards (Fig. 5B; P < 0.05). The results showed that gradual decreases in the BV/TV and Tb.Th were associated with gradual increases in the Tb.N and Tb.Sp over time.

#### Histochemical tartrate-resistant acid phosphatase (TRAP) staining

In order to evaluate whether VEGF can induce TMJ OA-like changes in the subchondral bone, immunohistochemical staining for TRAP was performed in the specimens at weeks 1 and 2. In the control and sham groups, very few TRAP-positive osteoclasts were identified in the condylar subchondral bone at weeks 1 and 2. However, in the vegf group, a high number of TRAP-positive osteoclasts, clustered in separate subchondral areas, was found at weeks 1 and 2 (Fig. 5C), indicating bone resorption. The number of TRAP-positive osteoclasts was significantly higher in the vegf group than in the control and sham groups (Fig. 5D; P < 0.05).

### Quantitative analysis of VEGF receptor 2 (VEGFR2)

**Figure 6:**
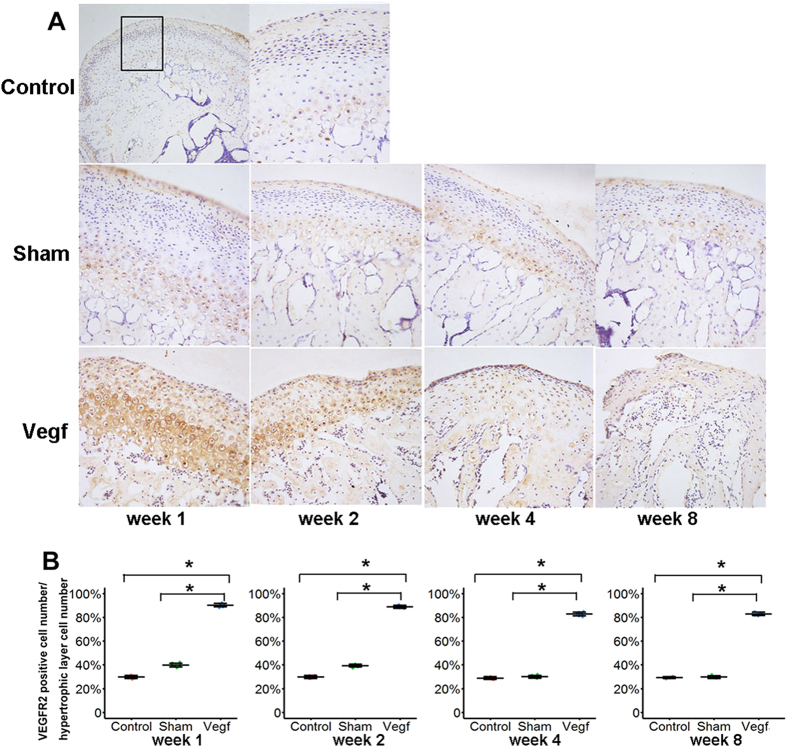
Expression of vascular endothelial growth factor receptor 2 (VEGFR2) in the condyle cartilage in the control, sham, and vegf groups at weeks 1, 2, 4, and 8. (**A**) Histological analysis of VEGFR2-positive chondrocytes. VEGFR2-positive chondrocytes are distributed in all cartilage layers in the vegf group from week 1 onwards. (**B**) Comparison of the percentage of VEGFR2-positive chondrocytes in the hypertrophic layer between the groups. The percentage of VEGFR2-positive chondrocytes is significantly higher in the vegf group than in the control and sham groups at all time points (*P < 0.05).

In the control group, VEGFR2-positive chondrocytes were sparsely distributed in the hypertrophic layer. In the sham group, there was a slight increase in the number of VEGFR2-positive chondrocytes at week 2, and these cells were localized to the hypertrophic layer, as in the control group. However, in the vegf group, VEGFR2-positive chondrocytes were distributed in all the cartilage layers from week 1 onwards (Fig. 6A). The percentage of VEGFR2-positive chondrocytes was significantly higher in the vegf group than in the control and sham groups at all time points (Fig. 6B; P < 0.05). At weeks 4 and 8, the number of VEGFR2-positive chondrocytes showed a slight decrease in the vegf group, but the differences between the groups remained significant.

### VEGF promoted receptor activator of nuclear factor-kappa-B ligand (RANKL)-induced osteoclast differentiation *in vitro*

**Figure 7:**
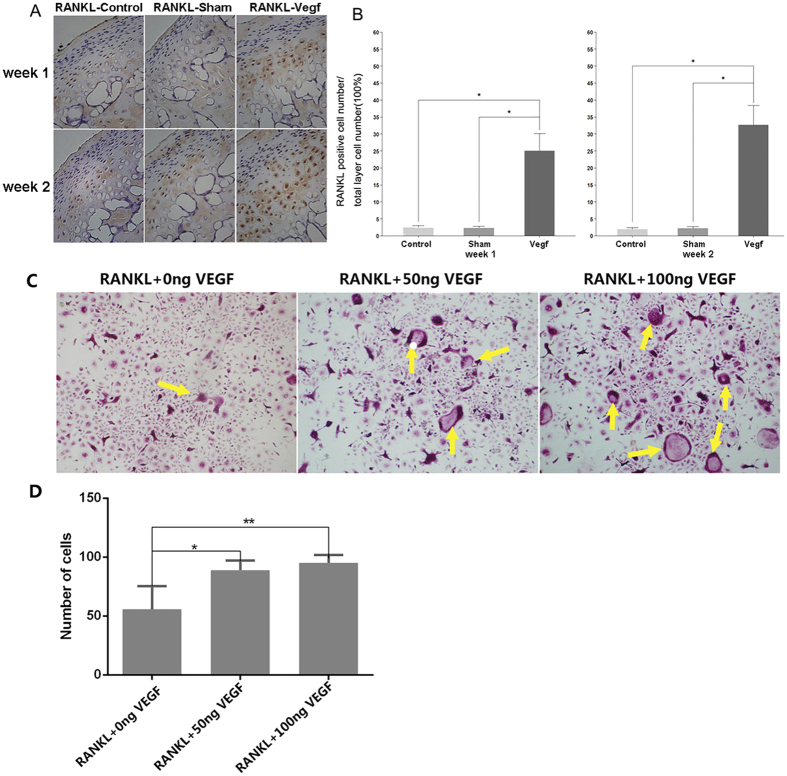
VEGF promoted receptor activator of nuclear factor-kappa-B ligand (RANKL)-induced osteoclast differentiation. (**A**) Immunohistochemical staining for RANKL at weeks 1 and 2. A high expression of RANKL is seen in the vegf group. (**B**) Comparison of the percentage of RANKL-positive cells between the groups. The percentage of RANKL-positive cells is significant higher in the vegf group than in the control and sham groups (*P < 0.05). (**C**) Bone marrow macrophages are stimulated with 30 ng/mL macrophage colony-stimulating factor, 50 ng/mL RANKL, and vascular endothelial growth factor (VEGF) (0, 50, or 100 ng). The number of tartrate-resistant acid phosphatase (TRAP)-positive cells increased in a dose-dependent manner (yellow arrow). (**D**) The number of TRAP-positive cells increased from 57.2 ± 3.3 cells/well (VEGF 0 ng) to 84.9 ± 5.6 cells/well (VEGF 50 ng) and 92.5 ± 4.8 cells/well (VEGF 100 ng) (*P < 0.05).

Immunohistochemical staining for RANKL was performed in specimens at weeks 1 and 2 in order to evaluate the effect of VEGF on the subchondral bone. In the control and sham groups, few RANKL-positive chondrocytes were identified within the cartilage. However, in the vegf group, many RANKL-positive chondrocytes were identified (Fig. 7A). The number of RANKL-positive chondrocytes was significantly higher in the vegf group than in the control and sham groups (Fig. 7B). The results suggested that VEGF could induce the expression of RANKL, which causes the destruction of subchondral bone.

To further investigate the role of VEGF in osteoclastic bone resorption, we treated bone marrow macrophages (BMMs) with both VEGF and RANKL *in vitro*. The BMMs were treated with RANKL and macrophage colony-stimulating factor in the presence of 0, 50, or 100 ng of VEGF. The number of TRAP-positive multinucleated osteoclasts increased in a dose-dependent manner (Fig. 7C). The number of TRAP-positive cells increased from 57.2 ± 3.3 cells/well (VEGF 0 ng) to 84.9 ± 5.6 cells/well (VEGF 50 ng) and 92.5 ± 4.8 cells/well (VEGF 100 ng) (Fig. 7D). These results showed that VEGF could effectively stimulate osteoclastogenesis.

## Discussion

The present study found that intra-articular injection of exogenous VEGF induced progressive osteoarthritic changes in the TMJ in mice. The typical changes of TMJ OA include cartilage degeneration and subchondral bone resorption. In the present study, VEGF-injected mice displayed loss of proteoglycan and a decrease in cartilage thickness, accompanied with irregular arrangement of chondrocytes and an increase in local cell-free areas within the condylar cartilage. In addition, subchondral bone destruction and resorption were confirmed using micro-CT analysis in VEGF-injected mice, with an increase in the number of osteoclasts in the early stage. These changes in cartilage and subchondral bone in VEGF-injected mice recapitulated the typical progression of TMJ degeneration over time, and the results were in accordance with those of previous studies on typical TMJ OA-like lesions^14^,^15^,^16^.

In our study, in the VEGF-injected mice, cartilage degeneration and metabolic changes in the cartilage were initially observed, and loss of proteoglycan, a decrease in cartilage thickness, and irregular arrangement of chondrocytes were gradually observed over time, suggesting irreversible destruction of the cartilage. Additionally, in the VEGF-injected mice, the Mankin score^17^ gradually increased with time, indicating progressive osteoarthritic changes in the condyles.

The present study found significantly higher numbers of MMP9-, and MMP13 -positive chondrocytes in the hypertrophic layer in the VEGF-injected mice than in the control and sham mice at weeks 1 and 2. Additionally, the number of apoptotic chondrocytes was higher in the VEGF-injected mice than in the control mice. Moreover, the number of VEGFR2-positive chondrocytes was significantly higher in the VEGF-injected mice than in the control and sham mice from week 1 onwards, which is consistent with the finding of our previous study that the expression of VEGFR-2 mRNA was high in the synovium of rabbit TMJs with internal derangement^17^. These results indicated that VEGF could induce the development and progression of cartilage degeneration in the TMJ. Additionally, VEGF might increase the expression of MMP-9 and MMP-13 in chondrocytes and induce chondrocyte apoptosis through VEGFR2, thus resulting in cartilage degeneration.

Subchondral bone destruction is an important feature of TMJ OA^18^. In our study, continuous subchondral bone loss was noted in the VEGF-injected mice. Local lesions were first detected 1 week after VEGF injection, using Micro-CT scanning. A high expression of RANKL was observed only in the cartilage of VEGF-injected mice, and RANKL is known to induce the destruction of subchondral bone^19^,^20^. Immunohistochemical analysis of TRAP showed a high number of osteoclasts, clustered in separate subchondral areas, in the first 2 weeks, indicating bone resorption. However, as time progressed, bone lesions, accompanied with local sclerosis, were observed in most of the VEGF-injected mice. These findings suggested that both bone resorption and remodeling were taking place within the subchondral bone. Moreover, a low BV/TV and Tb.Th, and a high Tb.N and Tb.Sp were noted in the VEGF-injected mice over time. Bone sclerosis and changes in the microstructure of the trabecula are characteristic of late-stage OA. The findings were similar to those for TMJ OA reported by Zarb *et al.*^21^.

RANKL-induced osteoclast differentiation has been confirmed in a previous study^22^. On treating BMMs with both VEGF and RANKL *in vitro*, we found that the number of osteoclasts increased with the increase in the VEGF dose, indicating that VEGF can induce bone destruction and resorption directly.

Many studies have shown that VEGF is associated with the development of OA in the TMJ^13^,^23^ and other joints^24^,^25^. However, to our knowledge, the present study is the first to demonstrate that VEGF can initiate TMJ OA. Thus, our study provides an animal model of TMJ OA that can be used to explore the molecular mechanisms of TMJ OA.

In conclusion, we successfully evaluated the effects of intra-articular injection of exogenous VEGF in the TMJ in mice and found that VEGF can initiate TMJ OA.

## Materials and Methods

### Ethics statement

Animal care and experiments were performed in accordance with protocols approved by the Animal Care and Use Ethics Committee of Shanghai Jiao Tong University School of Medicine (Number: 2013-55).

### Animals and experimental design

Forty-eight 10- to 12-week-old male Sprague-Dawley mice (provided by the Animal Experiment Laboratory of Shanghai Jiao Tong University School of Medicine) were used in this study. The mice were housed under specific pathogen-free conditions and were provided access to conventional chow and tap water ad libitum. All surgery was performed under chloral hydrate anesthesia, and all efforts were made to minimize suffering.

The animals were divided into the following 3 groups: a vegf group, sham group, and control group. In the vegf group, 16 mice (32 joints) received a 50-μL intra-articular injection (see injection procedure below) of a VEGF165 (PeproTec, Rocky Hill, NJ) solution (0.05 mg/mL VEGF165) once a week over a period of 4 weeks according to the study by Ludin *et al.*^26^. In the sham group, 16 mice (32 joints) received an injection of 50-μL saline into the upper compartment of the TMJ. The remaining 16 mice (32 joints) were not subjected to any treatment and were used as controls. Twelve mice (4 mice [8 joints] per group) were sacrificed at 1, 2, 4, and 8 weeks after the first injection, with an overdose of anesthesia solution by intraperitoneal injection, and the sacrificed mice were analyzed.

### VEGF administration

Mice were anesthetized using 10% chloral hydrate (300 mg/kg) by intraperitoneal injection. The fur on the preauricular region was shaved, and the exposed region was sterilized. After local injection of 2% lidocaine (1.5 mL), a preauricular incision was made to expose the TMJ capsule. A microinjection needle was used to inject 50-μL saline or VEGF165 solution into the upper compartment of the TMJ.

### Micro-CT analysis of the subchondral bone

After sacrifice, the TMJs of the mice were dissected for micro-CT analysis. The microstructural changes in each specimen were evaluated using a high-resolution, micro-CT system (GE eXplore Locus SP, London, ON, Canada). Scans were performed at 80 kV and 500 μA, and the spatial resolution was 5 μm. Parameters, including the BV/TV, Tb.N, Tb.Th, and Tb.Sp, were used for analysis of the trabecular microstructure.

### Tissue preparation and histological staining

TMJ specimens were fixed, decalcified, dehydrated, and embedded using conventional methods. HE, toluidine blue, and safranin-O and fast green staining were performed according to standard protocols for analysis under a light microscope. HE staining was used to assess condylar changes. Toluidine blue, and safranin-O and fast green staining were performed to determine proteoglycan changes. The thicknesses of the whole condylar cartilage and the hypertrophic layer were measured using the image analysis software NIS Elements D (Nikon, Tokyo, Japan). A modified Mankin scoring system^27^ was used to assess the osteoarthritic state of the articular cartilage. The scoring of the articular cartilage was based on pericellular and background safranin-O and fast green staining, chondrocyte arrangement, and the structural condition of the cartilage. The score for normal articular cartilage is 0, and the maximum score for degenerative articular cartilage is 10 (Table 1).

### Immunohistochemistry

TRAP staining was performed for the identification of osteoclasts, according to the manufacturer’s instructions (Sigma 387-A, St Louis, MO). A standard, 3-step, avidin-biotin complex immunohistochemical staining protocol was used. The primary antibodies were anti-VEGFR2 (CST 2472, Danvers, USA, 1:50 dilution), anti-RANKL (ab-9957; 1:50 dilution), anti-MMP9 (ab-38893; 1:50 dilution), and anti-MMP13 (ab39012, 1:50 dilution). Except anti-VEGFR2, all obtained from Abcam Biotechnology (MA, USA). The distribution of apoptotic chondrocytes was assessed using TUNEL staining (Roche, Mannheim, Germany). The number of osteoclasts was counted in the mineralized layer subjacent to the hypertrophic cell layer of the condylar cartilage in the TRAP staining specimens. TRAP-positive cells with 2 or more nuclei were counted as osteoclasts. The percentage of immuno-positive cells in other immunohistochemical staining specimens was calculated using NIS Elements D (Nikon) in 3 areas measuring 400 μm × 200 μm from the anterior, middle, and posterior regions of the condylar cartilage.

### Cell culture and osteoclast differentiation

Four to six-week-old C57BL/6 mice were used for cell culture. BMMs were isolated from whole bone marrow^28^,^29^. Cells were isolated from the femoral and tibial bone marrow and were cultured in T75 flasks with a-MEM supplemented with 10% FBS, 1% penicillin/streptomycin, and 10 ng/mL macrophage colony-stimulating factor for 24 h. Non-adherent cells were removed, and the adherent cells were cultured in a 5% CO_2_ incubator at 37 °C for 3–4 days until the cells were fully confluent. The BMMs were then seeded into a 96-well plate at a density of 9 × 10^3^ cells/well in complete a-MEM supplemented with 30 ng/mL macrophage colony-stimulating factor, 50 ng/mL RANKL, and different concentrations of vegf (0, 50, or 100 ng/mL). Cell culture media were replaced every 2 days until mature osteoclasts were obtained. The cells were then washed twice with phosphate-buffered saline, fixed with 4% paraformaldehyde for 20 min, and stained for TRAP using a Diagnostic Acid Phosphatase kit (Sigma). TRAP-positive cells with more than 3 nuclei were counted under a microscope.

### Statistical analysis

The Kruskal-Wallis nonparametric test was used to compare more than 2 groups of variables, and significance was set at P ≤ 0.05. When differences were found, the Mann-Whitney nonparametric test was used to confirm significant differences between 2 groups of variables, using the Bonferroni correction to set P-values. All statistical analyses were performed using the SPSS software package, version 16.0 (SPSS, Chicago, IL).

## Additional Information

**How to cite this article**: Shen, P. *et al.* Injecting vascular endothelial growth factor into the temporomandibular joint induces osteoarthritis in mice. *Sci. Rep.* **5**, 16244; doi: 10.1038/srep16244 (2015).

## Figures and Tables

**Table 1:** The modified Mankin scoring system used to evaluate the articular cartilage in mice.

1) Pericellular Safranin-O staining	
a. Normal	0
b. Slightly enhanced	1
c. Intensely enhanced	2
2) Background Safranin-O staining	
a. Normal	0
b. Slight increase or decrease	1
c. Severe increase or decrease	2
d. No staining	3
3) Arrangement of chondrocytes	
a. Normal	0
b. Appearance of clustering	1
c. Hypocellularity	2
4) Cartilage structure	
a. Normal	0
b. Fibrillation in the superficial layer	1
c. Fibrillation beyond the superficial layer	2
d. Missing articular cartilage	3
